# Music Preferences and Personality in Brazilians

**DOI:** 10.3389/fpsyg.2018.01488

**Published:** 2018-08-21

**Authors:** Lucia Herrera, João F. Soares-Quadros, Oswaldo Lorenzo

**Affiliations:** ^1^Department of Developmental and Educational Psychology, University of Granada, Melilla, Spain; ^2^Department of Music, Federal University of Maranhão, São Luís, Brazil; ^3^Department of Music Education, University of Granada, Melilla, Spain

**Keywords:** music preference, personality, Brazil, age, gender

## Abstract

This article analyzes the relationship between musical preference and type of personality in a large group of Brazilian young and adult participants (*N* = 1050). The study included 25 of 27 states of Brazil and individuals aged between 16 and 71 years (*M* = 30.87; *SD* = 10.50). Of these, 500 were male (47.6%) and 550 were female (52.4%). A correlational study was carried out applying two online questionnaires with quality parameters (content-construct validity and reliability), one on musical preference and the other on personality. The results indicate four main findings: (1) the musical listening of the participants is limited to a reduced number of styles, mainly Pop music and others, typical of Brazilian culture; (2) the Brazilian context supposes a determining aspect in the low preference of non-Brazilian music; (3) there is a positive correlation between most personality types analyzed and the Latin, Brazilian, Classical and Ethnic musical styles. A negative correlation between these types of personality and the consumption of Rock music was also observed; (4) musical preferences are driven not only by personality but in some cases they are also driven by socio-demographic variables (i.e., age and gender). Likewise, this work shows how participants make use of music in personality aspects that may be of interest for the analysis of socio-affective behavior (personality) as well as according to different socio-demographic variables (e.g., age and gender). More cross-cultural research on musical preference and personality would need to be carried out from a global perspective, framed in the context of social psychology and studies of mass communication.

## Introduction

The study of using music in everyday life and of musical preferences has been widely addressed in the scientific literature, in the last decades (Schäfer and Sedlmeier, 2009; Lonsdale and North, 2011; Schäfer et al., 2013). Regarding this subject, researchers have adopted in particular two different methodologies, so that listeners may indicate their musical preference: verbal preference/self-report assessment or sound preference/excerpt-based assessment (Fricke and Herzberg, 2017; Upadhyay et al., 2017).

The first method is more recurrent in the literature and consists of requesting participants to evaluate their musical preference from a list of music or of music styles (e.g., Rentfrow and Gosling, 2003; Gouveia et al., 2008; Lorenzo et al., 2014; Upadhyay et al., 2017). The second, however, differs from the first, since it uses fragments of music pieces and request participants to value their preference for each fragment heard (e.g., Rentfrow et al., 2011, 2012; Langmeyer et al., 2012; Greenberg et al., 2016).

According to Bonneville-Roussy et al. (2013), regardless of the methodology used, different investigations converged on two questions about musical preferences. Firstly, there is an underlying structure and, secondly, those are linked to different psychological characteristics.

Regarding its structure, it is common to find that different genres or music styles are grouped into a series of dimensions or factors. Thus, Rentfrow and Gosling (2003) obtained four dimensions of music preference: Reflective and Complex (Blues, Jazz, Classic, and Folk music), Intense and Rebellious (Rock, Alternative, and Heavy metal music), Upbeat and Conventional (Country, Soundtracks, Religious, and Pop music), and Energetic and Rhythmic (Rap/Hip-Hop, Soul/Funk, Electronic/Dance music). This classification has been used in different studies, although its structure has not always been confirmed (Langmeyer et al., 2012).

Other studies suggest five dimensions of music preference, identified by the acronym MUSIC: Mellow (e.g., Pop), Unpretentious (e.g., Country), Sophisticated (e.g., Classic), Intense (e.g., Rock), and Contemporary (e.g., Rap) (Rentfrow et al., 2011, 2012; Bonneville-Roussy et al., 2013; Fricke and Herzberg, 2017). However, the music styles included in each dimension may differ according to the study. Thus, it is possible to obtain even more dimensions. Hence, in the study published by Schäfer and Sedlmeier (2009), there are six dimensions: Sophisticated, Electronic, Rock, Rap, Pop, and Beat, Folk and Country Music. On the other hand, George et al. (2007) identified eight dimensions: Rebellious, Classical, Rhythmic and Intense, Easy Listening, Fringe, Contemporary Christian, Jazz and Blues, and Traditional Christian). North (2010) indicates ten dimensions: Classic Music, Jazz, Mainstream, Folk, Alternative Rock, Latin, Black Music, Dance, Rock, and Functional). The main reason for which the number of dimensions varies between different studies relies on the number of music styles taken into account and the cultural specificities of each study were identified. However, Rentfrow et al. (2011) highlighted the existence of three recurrent factors in the literature: a first factor defined by Classic and Jazz music styles. A second factor includes the Rock and Heavy Metal; and the third factor is defined by Rap and Hip-Hop.

Regarding the psychological characteristics associated with musical preferences, personality has been one of the most widely studied psychological correlates (e.g., Chamorro-Premuzic et al., 2010; Brown, 2012; Neuman et al., 2016). The model of personality on which many studies were based has been the Big Five model (Goldberg, 1992; Goldberg et al., 2006), which indicates that personality is structured around five domains: Extraversion (talkativeness, assertiveness, and energy), Agreeableness (good-naturedness, cooperativeness, and trust), Conscientiousness (orderliness, responsibility, and dependability), Emotional Stability (the polar opposite of Neuroticism), and Intellect/Imagination (originality, curiosity, and ingenuity), also known as Openness. Emotional reactions to music are determined by the personality of the listeners, a reason for which the study of personality in music preferences is relevant (Juslin et al., 2011).

In particular, the Openness domain appears as the best predictor of music preferences and tends to be associated with intense emotions prior music (Langmeyer et al., 2012; Liljeström et al., 2013). Different studies have proven that this personality trait correlates positively, especially with music styles considered sophisticated (e.g. Jazz, Blues and Classic) and rebellious/intense (e.g., Rock, Alternative, Punk, and Heavy metal) (Rentfrow and Gosling, 2003; George et al., 2007; Delsing et al., 2008; Langmeyer et al., 2012; Bonneville-Roussy et al., 2013; Fricke and Herzberg, 2017), presenting negative correlations with Pop, Soundtracks, and Gospel (Rentfrow and Gosling, 2003; Zweigenhaft, 2008; Langmeyer et al., 2012). Taking these as a whole, Jazz is the music style whose positive correlation is more reflected in the certain studies due to its positive correlation with Openness.

The Extraversion domain tends to correlate positively with Rap/Hip-Hop, Pop, and Soul/R&B/Funk styles (Rentfrow and Gosling, 2003; Delsing et al., 2008; Langmeyer et al., 2012; Bonneville-Roussy et al., 2013; Fricke and Herzberg, 2017). However, George et al. (2007) have found different results in relation to the first two styles, obtaining positive correlations with Openness and negative with Agreeableness and Conscientiousness. Agreeableness, in turn, often appears positively correlated with Gospel and Pop styles and, to a lesser extent, with Electronic, Rap/Hip-Hop, Soul/R&B/Funk, and Soundtracks (Rentfrow and Gosling, 2003; Delsing et al., 2008).

Furthermore, different studies agree on the negative correlation between the Conscientiousness domain and Alternative styles, Punk, and Rock and the positive correlation with Country music, Gospel, and Pop style (Rentfrow and Gosling, 2003; George et al., 2007; Bonneville-Roussy et al., 2013).

Regarding the domain or personality of Neuroticism trait, we observed that this correlates negatively with rebellious/intense styles (Alternative and Rock) (Langmeyer et al., 2012; Fricke and Herzberg, 2017) and positively with the Classic style (Delsing et al., 2008; Dunn et al., 2011).

The literature indicates that, in addition to personality, there are other variables that influence music preferences, that is, age and gender. On the first one, there are several studies corroborating that music preferences and their intensity are directly related to age (e.g., North, 2010; Fricke and Herzberg, 2017). At this regard, Levitin (2011) considers the period between 18 and 20 years as the time limit for the acquisition of new music preferences. This is the stage in which occurs the crystallization of the musical taste and, according to Delsing et al. (2008), it may be related to personal maturity and identity formation.

Zweigenhaft (2008) observed differences in the preference of listening to Punk, Blues, Country, and Soul/Funk styles, this being the most listened by adolescents and others adults. Soares de Quadros and Lorenzo (2010) found a higher frequency of listening among Brazilian students already aged 19 years old, when compared to the youngest for the Bossa-Nova, Bolero, Flamenco, Jazz, Salsa, and Soul styles. On the other hand, North (2010) found a positive correlation between age and preference for Classic, Jazz, Mainstream, Folk, Latin, and Functional metastyles. Negative association was observed for MOBO, Dance, Alternative Rock, and Rock metastyles. For the author, age is positively associated with the preference to listen more complex music styles. Coinciding with this idea, Lorenzo et al. (2014) found a greater preference for listening styles considered as musically less complex (Dance, Breakdance, Speed, and Trash) for the younger age group (16–19 years), when compared to adults.

In their study, Bonneville-Roussy et al. (2013) verified that while the preference for certain types of music increased with age (e.g., Intense, Contemporary), for others it was the opposite (e.g., Unpretentious, Sophisticated). In addition, they observed a singular behavior in preferring the Mellow music (e.g., Electronic/Dance), decreasing up to 30 years old and increasing again after 50 years old (Fricke and Herzberg, 2017).

Regarding the second variable, i.e., gender, there are several studies affirming the existence of a greater preference of men toward styles considered more “hard,” exciting, linked to non-conformity behaviors with imposed patterns and social rules, which strengthen the ties within the peer group (like Rap/Hip-Hop and Reggae). On the contrary, women are inclined to prefer “softer” music, with more emotional content, made to dance and with a clear dependence on more social media patterns, such as Pop (e.g., Pimentel et al., 2005; Colley, 2008; Gouveia et al., 2008; North and Hargreaves, 2008).

North (2010) verifies that men show a high preference for Jazz, Dance, Rock, and Functional metastyle, while women increasingly choose Classical, Mainstream, Folk, Alternative Rock, Latin, and MOBO styles. On the other hand, George et al. (2007) observed a greater preference of men toward Rebellious music (e.g., Heavy metal), while women mainly prefer Easy Listening styles (e.g., Pop). However, Bonneville-Roussy et al. (2013) find gender differences only for listening to Unpretentious music (e.g., Pop and Country), with a greater preference for the female gender.

Soares de Quadros and Lorenzo (2010) observed that, in general, Brazilian women listen to a wider variety of musical styles when comparing to men: Pagode, Sertanejo, Romantic, Forró, Axe music, Bolero, Pop, Jazz, Salsa, Ska, Soundtracks, and Gospel. This result is related to the hypothesis about the greater musical eclecticism of the female gender (Crowther and Durkin, 1982). Tipa (2015) highlights that women tend to use music as sound background in much of their daily activities, while for men this plays a major role especially in social and affective relationships established between peer groups. At this regard, Soares de Quadros and Lorenzo (2010) verify a greater inclination of male Brazilian adolescents to play music strengthening the ties within the same group, such as Heavy Metal and Rap.

For North and Hargreaves (2008), the main reason for a gender effects on musical listening behavior would be related to differences in the use of music. While men listen to music looking for the effect on social membership with their peer group, women listen to music as a way to satisfy their emotional needs. This is related to what Gouveia et al. (2008) exposed, concerning that women adhere more to normative values and have a greater tendency to emotional contagion, while men are more liberal and seekers of sensations. According to what mention above, North (2010) adds that men use music to be creative, awaken the imagination and pleasing friends. Women, on the other hand, use it for moments of pleasure, relieve boredom, express feelings and emotions and mitigate the loneliness.

According to what has been described so far, the main goal of this research is to analyze music preferences that young and adult Brazilians present as well as the relationship of these with their personality. This objective is articulated around three specific objectives. Firstly, perform an adaptation of different tests, allowing to measure personality and music preferences in Brazil. Secondly, analyze the correlation between these aspects. Thirdly, identify if musical preferences differ according to age and gender.

## Materials and Methods

### Participants

A non-probabilistic sampling was used. Participants were provided with access according to their location through a universe of people included in Facebook groups linked to different areas of knowledge of Brazilian universities. To access the questionnaire, it was necessary to have some relationship with University studies, either as bachelor or as graduate students; to live in Brazil; and to authorize the publication of the information obtained through research.

One thousand and sixty-eight people answered to the questionnaire. Once removed invalid cases (*n =* 18), the final sample consisted of 1050 Brazilians, 500 men (47.6%) and 550 women (52.4%), with a minimum age of 16 and maximum of 71 years old (*M_Age_* = 30.87; *SD_Age_* = 10.50). This leads the formation of three groups, attending the accumulated percentage, group of 16–24 years (*n* = 347), group of 25–33 years (*n* = 370), and group of 34–71 years (*n* = 333).

The participants lived in 212 cities belonging to 25 of the 27 States of Brazil, being the three largest involving São Paulo (20.1%), Minas Gerais (17.9%) and Maranhão (17.6%). Depending on geographical regions, it is shown in **Table 1** that the majority of the participants were from South-Eastern Brazil (50.0%).

**Table 1:** Participants’ distribution by geographical region in Brazil.

Regions	Frequency	%
Southeast	525	50.0
Northeast	340	32.4
South	121	11.5
Midwest	40	3.8
North	24	2.3
Total	1050	100.0


Regarding the level of training (see **Table 2**), 38.4% was pursuing undergraduate studies.

**Table 2:** Training level.

Level	Frequency	%
Graduation’s Degree in progress	403	38.4
Graduation’s Degree completed	166	15.8
Specialization in progress	51	4.9
Specialization completed	107	10.2
Master’s Degree in progress	71	6.8
Master’s Degree completed	87	8.3
PhD in progress	79	7.5
PhD completed	86	8.2
Total	1050	100.0


### Instruments

To collect information, using the *Google Docs* platform, a template was designed. To include a brief introduction about the main objective of the research and how the designed instrument was structured online, it consisted of four sections: Identification data, Personality Questionnaire, Questionnaire on Preference of Musical Styles Listening and Authorization. Next, each item is described.

#### Identification Data

This section was designed to collect descriptive data of the participants, e.g., for example gender, age, city, and state, as well as education level, among others.

#### Personality Questionnaire

Based on the concept of personality measurement focused on the International Personality Item Pool (IPIP) as a prototype (Goldberg et al., 2006), the *Questionnaire for Administering the 50-Item Set of IPIP Big-Five Factor Markers*, from the IPIP Web site at http://ipip.ori.org/, was used. *IPIP Big Five Factor Markers* (Goldberg, 2001) is formed of 50 items structured around five scales based on study developed by Goldberg (1992): Extraversion, Agreeableness, Conscientiousness, Emotional Stability, and Intellect/Imagination (also known as Openness). The participants have to answer on a five points Likert scale, where 1 = Very inaccurate and 5 = Very accurate.

For the study, an adapted version of the test to Portuguese was used, following the established criteria by the International Test Commission (2005). These criteria have been widely used in tests adaptation (Muñiz et al., 2013). In this adaptation, four Brazilian psychologists, fluent in English, participated. They made the linguistic and cultural adaptation of the test, using a direct and inverse translation of the original instrument. Subsequently, a pilot test was carried out (*n* = 11) to obtain feedback on their understanding and, in this way, to be able to make final adjustments.

The construct validity and reliability of the questionnaire were calculated. Thus, construct validity was analyzed using a data reduction technique. The extraction method was the principal component analysis (PCA) and the rotation method the Varimax with Kaiser Normalization. Firstly, the feasibility of carrying out this analysis was determined and confirmed by values obtained in measuring Kaiser-Meyer-Olkin sampling adequacy, *KMO* = 0.864, and the Bartlett sphericity test, *χ^2^* = 16505.779, *p* = 0.000. The analysis identified five factors that explained the 39.45% of the variance. Of the 50 items, 88% loaded as expected based on the theoretical framework (see **Supplementary Table S1**). All items on the Extraversion (E) scale loaded most highly on the same factor. Likewise, the items on the Intellect scale (I). However, on the E scale, item 16 saturated below 0.30 as well as item 45 on I scale. Of the items on the Agreeableness scale (A), two items scored higher on the Emotional Stability scale (“Insult people” and “Have a soft heart”) and one on the Extraversion scale (“Make people feel at ease”). In addition, item 2 saturated with values below 0.30. Regarding the items of the Conscientiousness scale (C), all were included in the same factor except one (“Pay attention to details”), which saturated higher in the Intellect scale. Similarly, items 13 and 18 saturated below 0.30. On the Emotional Stability (ES) scale, the exceptions were items “Am relaxed most of the time” and “Worry about things,” which saturated with a higher value on Conscientiousness and Agreeableness scales respectively. The rest of the items were included in this factor. However, items 9 and 14 saturated below 0.30.

The aforementioned led to take the decision to delete those items that saturated below 0.30 in the factor that theoretically should make it (items 2, 9, 13, 14, 16, 18, and 45) as well as those that had a greater effect in factors different from those that theoretically corresponded to (items 12, 27, and 47).

In the second analysis, the Kaiser-Meyer-Olkin sample adequacy measurement was 0.847, and the Barlett’s sphericity test *χ^2^* = 13764.989, *p* = 0.000. Five factors were identified and these explained 44.23% of the variance (see **Supplementary Table S2**). The first factor (ES) explained 11.543% of the total variance, the second (E) 8.435%, the third (I) 8.352%, the fourth (C) 8.135% and the fifth (A) 7.764%.

The reliability of the questionnaire was calculated using the *Cronbach’s alpha* internal consistency index. The total value was *α* = 0.739.

#### Questionnaire on the Preference of Listening to Musical Styles

An adaptation of the *Questionnaire on Musical Style Preferences* (Cremades et al., 2010) was used, specifically in the first part, the one concerning the frequency of listening to musical styles. In the initial questionnaire, this section included 49 different musical styles and participants needed to indicate their frequency of listening according to a five points Likert scale (1 = Never, 5 = Always).

The adaptation of the questionnaire to Portuguese language included new musical styles, which were listened in Brazil. This resulted in a final questionnaire including 73 musical styles (see **Supplementary Table S3**). A response option opened to others musical styles, which were heard but not previously collected, was even added. This task was accomplished through the collaboration of three experts of Brazilian music, fluent in Spanish and necessary due to the linguistic and cultural adaptation of the questionnaire as well as the validation of content by experts’ opinion that followed the criteria established by Barbero (2006). In response options, along with the original questionnaire, the option “I do not know that music style. I cannot appreciate it” was included. The questionnaire was also applied to a pilot test, in which the individuals that carried out the previous test participated to this, aiming at ensuring their correct understanding.

Construct validity was determined, as in the previous questionnaire, through a data reduction technique. The principal component analysis (PCA) was followed as extraction method. Also, Varimax with Kaiser Normalization was the rotation method used. Both the results of sample adequacy by Kaiser-Meyer-Olkin measurement, *KMO* = 0.927, and Barlett’s sphericity test, *χ^2^* = 47761.543, *p* = 0.000, indicated the suitability of performing this type of analysis. We found that 11 factors explained 61.232% of the total variance (see **Supplementary Table S4**), which are detailed below.

##### Factor 1 (11.751% of the variance)

This factor included two groups of music styles: Classic and Ethnic. Thus, for example, Baroque and Romantic Music were included in the first group, while Ethnic Music and Tango, among others, were included in the second group.

##### Factor 2 (8.385%)

It included different musical styles, grouped in the Rock Music category (for example, Hard Rock and Heavy Metal).

##### Factor 3 (8.309%)

It referred to more popular Brazilian music styles such as, for example, Arrocha and Pagode, and therefore named as Brazilian Mainstream Music.

##### Factor 4 (6.070%)

This fourth factor included Latin music to dance, as Merengue and Mambo, defined as Latin Dance Music.

##### Factor 5 (5.443%)

This was more referred to highly sophisticated styles of Brazilian Music such as, for example, Brazilian Popular Music and Bossa-Nova.

##### Factor 6 (4.612%)

It included different music styles related to Electronic Music (for example, Dance and Electronic).

##### Factor 7 (4.040%)

The musical styles of this factor were characterized according to their American origin, and therefore named as American Music. As an example, Soul and Jazz were included.

##### Factor 8 (3.437%)

It was structured around Alternative Music (for example, Indie).

##### Factor 9 (3.262%)

It included Music with Afro-American origin (Rap and Reggae, among others).

##### Factor 10 (3.092%)

This factor related to Pop Music.

##### Factor 11 (2.831%)

Upbeat Music brought together musical styles that are frequently associated with positive emotions (for example, Gospel and Instrumental Music and/or Movie Soundtracks).

**FIGURE 1:**
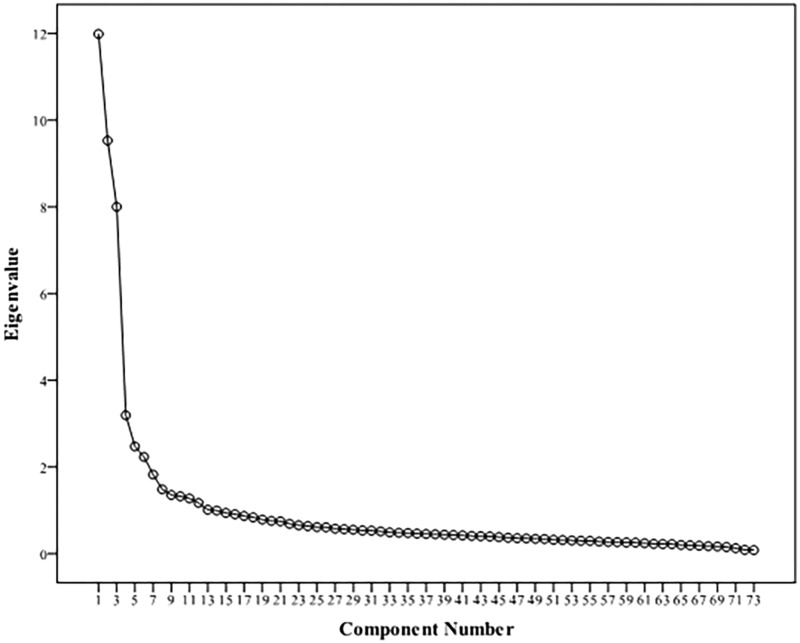
Scree plot (Principal Component Analysis).

Examination of the scree plot (see **Figure 1**) suggested an “elbow” at roughly seven factors. Thus, there are conflicting results from the eigenvalue approach and the scree plot approach. That is why a parallel analysis (Monte Carlo simulation) was developed to determine how many components this method suggests to retain (O’Connor, 2000; Green et al., 2015). Parallel analysis suggested nine factors above chance (see **Supplementary Table S5**).

Due to the aforementioned, it was decided to retain nine components. The first five factors coincide with the MUSIC model, according to the classification of Rentfrow et al. (2011, 2012): Factor 1 = Sophisticated; Factor 2 = Intense; Factor 3 = Mellow; Factor 4 = Contemporary; and Factor 5 = Unpretentious. On the other hand, the last four are included in other classifications such as North’s (2010).

Finally, the total reliability of the questionnaire was *α* = 0.915.

### Procedure

#### Information Collection

An online template was created in *Google Docs* and contained the two questionnaires used ^1^. This template was available on the Web, for 6 months. The study and possibility to participate in the social network *Facebook*, through aforementioned networks and academic groups, was reported.

The authorization of the participants to use the results obtained exclusively for educational and scientific purposes was requested.

#### Statistical Analysis of the Data

The statistical program *IBM SPSS Statistics 23* was used to carry out the statistical analysis.

When the psychometric characteristics of the two instruments used were guaranteed, it was determining if data complied with a normal distribution. The aim was to take decisions regarding the use of parametric or non-parametric tests. For this, the Kolmogorov–Smirnov test was used in two measuring instruments. The results were significant (*p* < 0.05), so the null hypothesis regarding the Gaussian distribution of the data was rejected. Likewise, it was assessed if the requirement of homoscedasticity or equality of variances between the comparison groups was met. Regarding the gender, Levene statistic was used through the Student’s *T*-test for independent samples. Thus, in *IPIP Big Five Factor Markers* not all factors fulfilled the equality of variances, according to the gender. On the other hand, regarding the three age groups, Levene statistic was also used, although via the Analysis of Variance, thus determining that the equality of variances was not met in all cases (*p* < 0.05).

Based on what was previously described, non-parametric tests were performed. At this regard, the Spearman correlation test was used to analyze a possible relationship between the five scales of the *IPIP Big Five Factor Markers* and the eleven factors found in the *Questionnaire on Musical Style Preferences*. In order to determine if there were differences according to the gender, the Mann–Whitney *U* test was used, while the Kruskal–Wallis *H* test was used for the analysis conducted according to the age group. Finally, a hierarchical regression analysis was carried out to determine the incremental validity of socio-demographic variables (i.e., age and gender) and personality in the prediction of musical preferences.

## Results

### Frequency of Listening to Music Styles

**Table 3** shows the frequency of listening to different groups of music styles or music dimensions. The two excluded factors after the parallel analysis have also been included in order to determine their listening frequency. However, they will not be used for subsequent statistical analyzes.

**Table 3:** Frequency of listening to musical dimensions.

Musical dimensions	Mean	Median	Mode
Classical and Ethnic Music	2.86	2.80	2.00
Rock Music	2.70	2.50	2.00
Brazilian Mainstream Music	2.62	2.45	2.00
Latin Dance Music	2.33	2.17	2.00
Brazilian Music	3.69	3.67	3.00
Electronic Music	2.48	2.43	2.00
American Music	3.08	3.00	3.00
Alternative Music	2.72	2.67	2.00
Afro-American Music	2.77	2.50	2.00
Pop Music	4.03	4.00	4.00
Upbeat Music	3.49	3.50	3.00


In **Table 3**, it can be observed that Pop Music is the most listened (*Mdn* = 4.00). It followed Brazilian Music (*Mdn* = 3.67), Upbeat Music (*Mdn* = 3.50) and American Music (*Mdn* = 3.00). On the other hand, a lower preference appears in Latin Dance Music (*Mdn* = 2.17) and Electronic Music (*Mdn* = 2.43) styles.

### Correlation Between Personality and Music Preference Dimensions

In order to determine to what extent different scales of personality relate to the 11 musical dimensions, a Spearman correlation analysis was implemented (see **Table 4**).

**Table 4:** Spearman correlation analysis of musical dimensions and personality scales.

Musical dimensions	Statistics	Personality				
		E	A	C	ES	I
Classical and Ethnic Music	*Rho*	0.068*	0.028	0.135***	0.108***	0.232***
	*p*	0.027	0.369	0.000	0.000	0.000
Rock Music	*Rho*	-0.033	-0.071*	-0.148***	-00.095**	0.137***
	*p*	0.283	0.021	0.000	0.002	0.000
Brazilian Mainstream Music	*Rho*	0.204***	0.068*	0.042	0.052	-0.100**
	*p*	0.000	0.028	0.169	0.093	0.001
Latin Dance Music	*Rho*	0.179***	0.069***	0.166***	0.182***	0.097**
	*p*	0.000	0.025	0.000	0.000	0.002
Brazilian Music	*Rho*	0.189***	0.130***	0.133***	0.145***	0.088*
	*p*	0.000	0.000	0.000	0.000	0.004
Electronic Music	*Rho*	0.091**	0.004	0.007	-0.045	0.091**
	*p*	0.003	0.904	0.928	0.147	0.003
American Music	*Rho*	0.131***	0.050	-0.048	0.070*	0.168***
	*p*	0.000	0.104	0.121	0.024	0.000
Alternative Music	*Rho*	0.023	0.001	-0.173***	-0.071*	0.130***
	*p*	0.463	0.967	0.000	0.022	0.000
Afro-American Music	*Rho*	0.138***	-0.011	-0.090**	0.030	-0.018
	*p*	0.000	0.732	0.004	0.328	0.567


*^∗∗∗^*p* < 0.001, ^∗∗^*p* < 0.01, ^∗^*p* < 0.05. E, Extraversion; A, Agreeableness; C, Conscientiousness; ES, Emotional Stability; I, Intellect.*

As one can see, group correlations were not high. However, Classic and Ethnic Music dimension obtained the highest correlation, in this case according to the Intellect personality scale, *ρ* = 0.232, *p* = 0.000. This is followed by the correlation between Brazilian Mainstream Music dimension and the Extraversion personality scale, *ρ* = 0.204, *p* = 0.000.

### Musical Preferences According to Age and Gender

First, the Kruskal–Wallis *H* Test was used to determine if the frequency of listening to different music dimensions varied according to the age group (see **Table 5**).

**Table 5:** Musical dimensions by age group (Kruskal–Wallis *H* Test).

Musical dimensions	Age group (Years)	*N*	Average rank	*χ^2^*	*p*
Classical and Ethnic Music	16–24	347	456.37	78.282***	0.000
	25–33	370	481.90		
	34–71	333	645.98		
Rock Music	16–24	347	568.19	24.131***	0.000
	25–33	370	544.78		
	34–71	333	459.60		
Brazilian Mainstream Music	16–24	347	500.64	4.670	0.097
	25–33	370	549.51		
	34–71	333	524.73		
Latin Dance Music	16–24	347	379.18	180.430***	0.000
	25–33	370	516.21		
	34–71	333	688.29		
Brazilian Music	16–24	347	420.91	99.838***	0.000
	25–33	370	510.34		
	34–71	333	651.33		
Electronic Music	16–24	347	506.99	2.091	0.352
	25–33	370	530.42		
	34–71	333	539.31		
American Music	16–24	347	486.26	11.552**	0.003
	25–33	370	526.64		
	34–71	333	565.13		
Alternative Music	16–24	347	593.37	27.325***	0.000
	25–33	370	503.60		
	34–71	333	479.11		
Afro-American Music	16–24	347	548.15	5.731	0.057
	25–33	370	532.37		
	34–71	333	494.27		


*^∗∗∗^*p* < 0.001, ^∗∗^*p* < 0.01.*

The older group used more than the rest music styles grouped in following dimensions: Latin Dance Music, *χ^2^* = 180.430, *p* = 0.000; Brazilian Music, *χ^2^* = 99.838, *p* = 0.000; Classic and Ethnic Music, *χ^2^* = 78.282, *p* = 0.000; and American Music, *χ^2^* = 11.552, *p* = 0.003. On the contrary, the younger age group was the one that most listened to Alternative Music, *χ^2^* = 27.325, *p* = 0.000; and Rock Music, *χ^2^* = 24.131, *p* = 0.000.

Secondly, it was analyzed if there were differences according to the gender through the Mann–Whitney *U* Test (see **Table 6**).

**Table 6:** Musical dimensions by gender (Mann–Whitney *U* Test).

Musical dimensions	Gender	*N*	Average rank	*U*	*p*
Classical and Ethnic Music	Male	500	575.01	112747.500***	0.000
	Female	550	480.50		
Rock Music	Male	500	580.50	109998.500***	0.000
	Female	550	475.50		
Brazilian Mainstream Music	Male	500	507.15	128324.500	0.061
	Female	550	542.18		
Latin Dance Music	Male	500	540.50	129999.000	0.123
	Female	550	511.86		
Brazilian Music	Male	500	499.69	124596.000**	0.008
	Female	550	548.96		
Electronic Music	Male	500	540.29	130105.500	0.131
	Female	550	512.06		
American Music	Male	500	545.13	127686.000*	0.045
	Female	550	507.66		
Alternative Music	Male	500	553.30	123602.000**	0.004
	Female	550	500.23		
Afro-American Music	Male	500	533.43	133533.500	0.416
	Female	550	518.29		


*^∗∗∗^*p* < 0.001, ^∗∗^*p* < 0.01, ^∗^*p* < 0.05.*

Men prefer to listen to Classic and Ethnic Music, *U* = 112747.500, *p* = 0.000; Rock Music, *U* = 109998.500, *p* = 0.000; Alternative Music, *U* = 123602.000, *p* = 0.004; and American Music, *U* = 127686.000, *p* = 0.045. On the other hand, women have a greater frequency of listening to Brazilian Music, *U* = 124596.000, *p* = 0.008.

### Incremental Validity of Socio-Demographic Variables and Personality

To determine the extent to which a musical style within the musical dimensions was driven by the socio-demographic variables (i.e., age and gender) or personality, a series of step-wise linear regressions were performed. First, nine regressions analyses were conducted in which the factor loadings of the musical dimensions were regressed onto the socio-demographic variables at step 1 and the personality at step 2. These analyses shed light on how much variance in the musical dimensions is accounted for by socio-demographic variables and whether personality adds incremental validity.

As can be seen in the top of **Table 7**, age and gender accounted for significant proportions of variance for each of the musical dimensions, with values for *R^2^* ranging from 0.004 for Brazilian Mainstream Music to 0.124 for Latin Dance Music. When the personality was added to the regression models, the amount of explained variance increased significantly for all nine of the nine musical dimensions. Specifically, adding personality to the regressions increased the *R^2^* values. These findings raise the question of whether personality accounts for more unique variance than do socio-demographic variables.

**Table 7:** Step-wise linear regressions of musical dimensions with socio-demographic variables and personality.

	CEM	RM	BMM	LDM	BM	EM	AM	ALM	AAM
Step 1. Age and Gender	0.084	0.055	0.004	0.124	0.098	0.005	0.015	0.040	0.006
Step 2. Personality	0.126	0.097	0.069	0.151	0.132	0.018	0.057	0.089	0.036
*Δ F*	10.005***	9.685***	14.612***	6.689***	8.032***	2.900*	9.351***	11.117***	6.468***
Step 1. Personality	0.066	0.065	0.068	0.061	0.067	0.014	0.047	0.065	0.030
Step 2. Age and Gender	0.126	0.097	0.069	0.151	0.132	0.018	0.057	0.089	0.036
*Δ F*	36.067***	18.841***	0.846	55.109***	38.844***	2.311	5.817**	13.589***	3.579*


*^∗∗∗^*p* < 0.001, ^∗∗^*p* < 0.01, ^∗^*p* < 0.05. CEM, Classical and Ethnic Music; RM, Rock Music; BMM, Brazilian Mainstream Music; LDM, Latin Dance Music; BM, Brazilian Music; EM, Electronic Music; AM, American Music; ALM, Alternative Music; AAM, Afro-American Music. Cell entries are *R^2^* values derived from step-wise linear regressions. *Δ F* = F change for each regression analysis.*

To address that question, another set of nine step-wise linear regression analyses were performed in which the factor loadings of the musical dimensions were regressed onto the personality at step 1 and then the socio-demographic variables at step 2. As can be seen in the bottom rows of **Table 7**, personality also accounted for significant proportions of variance, with values for *R^2^* ranging from 0.014 for Electronic Music to 0.068 for Brazilian Mainstream Music. However, socio-demographic variables also appear to account for significant proportions of unique variance, with significant increases in *R^2^* for Classical and Ethnic Music, *ΔF* = 36.067, *p* = 0.000; Rock Music, *ΔF* = 18.841, *p* = 0.000; Latin Dance Music, *ΔF* = 55.109, *p* = 0.000; Brazilian Music, *ΔF* = 38.844, *p* = 0.000; American Music, *ΔF* = 5.817, *p* = 0.003; Alternative Music, *ΔF* = 13.589, *p* = 0.000; and Afro-American Music, *ΔF* = 3.579, *p* = 0.028.

## Discussion

This research focused on the analysis of musical preferences and personality of Brazilian adults and young individuals. In line with previous studies (Langmeyer et al., 2012; Liljeström et al., 2013), it became clear that the personality scale related to Intellect/Openness is one related to all music preferences dimensions, with the exception of Afro-American Music in this study. On the one hand, it has been found that Intellect/Openness correlates positively with styles considered more complex musically (e.g., Classic and Ethnic Music), rebellious (e.g., Rock Music) and exciting (e.g., Electronic Music) (Rentfrow and Gosling, 2003; Vella and Mills, 2016). On the other hand, a negative correlation was identified with styles defined as light music, characterized by their emotional content, less musical complexity and high connection to the mass media (e.g., Brazilian Mainstream Music) (Zweigenhaft, 2008). Thus, it is possible that individuals presenting a greater development of the Intellect/Openness dimension tend to listen to highly artistic and aesthetic music styles, dynamic and exciting or capable to awake the curiosity to what is heard.

Similarly, the Agreeableness personality scale correlated positively with music styles emphasizing positive emotions in their listeners (e.g., Brazilian Music), which agrees with previous studies (Langmeyer et al., 2012; Bonneville-Roussy et al., 2013). In addition, the association of this personality trait with styles linked to Latin-American rhythms was observed, which favor couple or group dancing (e.g., Latin Dance Music). The results found herein indicate that listeners with a greater tendency toward Agreeableness appreciate music emphasizing an optimistic view of life, which is often heard and shared in social groups. On the other hand, a negative, although low, correlation was observed between Agreeableness and styles considered anti-authoritarian, e.g., Rock and its derivatives, results in line with George et al. (2007). However, North (2010) and Zweigenhaft (2008) affirm that Rock fans do not necessarily have a vision of the rebel world, contrary to part of the specialized literature. Due to the divergence of results found in this section, it is necessary to carry out further work, as this will allow deepening and finding explanatory hypotheses with greater convergence.

The results obtained for Conscientiousness converge with previous studies (Rentfrow and Gosling, 2003; Delsing et al., 2008), suggesting that individuals with a strong Conscientiousness trait have a greater preference for styles associated with positive, musically more complex emotions and that somehow favor introspection. On the other hand, in this study it was also found that Conscientiousness correlated negatively with musical dimensions associated with anti-authoritarian behavior (Rock Music, Afro-American Music, and Alternative Music). This confirms the outstanding characteristic of this type of listeners with respect to the value they attribute to certain aspects of their personality, e.g., responsibility and discipline.

Extraversion was the only dimension that did not present negative correlations with different musical dimensions evaluated. This may suggest that individuals with strong Extraversion traits possess omnivorous musical tastes, being more open to appreciation and musical cultural diversity (Elvers et al., 2015). Regarding the positive correlations found, firstly, it was observed the highest association with Brazilian Mainstream Music, a musical dimension that adds music styles of mass consumption, frequently present in parties and clubs of different Brazilian contexts, thus corroborating the results published by Langmeyer et al. (2012). Secondly, an association between the Extraversion personality scale and the music strongly characterized by its link with music styles favoring a collective musical practice (e.g., Brazilian Music and Latin Dance Music) was established. This might be related to one of the characteristics of this personality trait, namely gregariousness, which is defined as the need of the individual to share experiences (musical) with his social group (Lamers et al., 2012). As expected, Extraversion also appeared associated with music styles defined as energetic and rhythmic (e.g., Afro-American Music), a result in line with different works (Langmeyer et al., 2012; Fricke and Herzberg, 2017). For all what previously described, it is possible to assume that individuals with high Extraversion scores tend to prefer music valuing assertiveness, positive emotions and social exchange.

The Emotional Stability personality scale appears as the one with the lowest incidence, although the one providing the most divergent results when compared to the scientific literature. First, a negative correlation was found between this personality trait and music styles considered rebellious and linked to anti-authoritarian behavior (e.g., Rock Music). Thus, it showed divergence with the study by Langmeyer et al. (2012), but it was in agreement with Fricke and Herzberg (2017). Linking the results, we found and the features characterizing the opposite end to Emotional Stability, i.e., Neuroticism, it can be suggested that more neurotic individuals tend to prefer music often appearing in the literature as frequently associated with anti-social behaviors, e.g., alcohol and use of illicit drugs, violence, suicide and other (Lester and Whipple, 1996; Pimentel et al., 2009; Till et al., 2016). Secondly, positive correlations were found with more sociable music styles (e.g., Latin Dance Music), emphasizing positive emotions (e.g., Brazilian Music) that might be related to the fact that individuals more emotionally stable tend to be more friends one to another. They are also quiet and presenting little mood changes. On the other hand, Emotional Stability was also positively associated with more reflective and complex music styles (e.g., Classic and Ethnic Music), contrary to the results found by Delsing et al. (2008).

Regarding the analysis conducted according to variables, e.g., age and gender, it was observed that the results obtained for the age variable converge, to a large extent, with the study by North (2010). This indicates that older individuals prefer listening to Latin, more sophisticated and of greater music complex styles. However, young people showed a greater preference for music styles related to Rock music. In turn, these results are different from those found by Bonneville-Roussy et al. (2013). On gender, men were more interested in musically more complex styles (e.g., American Music), with critical content and associated with rebellious behavior (e.g., Rock Music) (George et al., 2007; North, 2010). On the other hand, women showed preference toward musical styles which emphasize positive emotions and are more framed into the Brazilian culture (Brazilian Music), these being results in agreement with previous studies (Pimentel et al., 2005; Colley, 2008).

Taken together, the results of the two groups of step-wise linear regressions carried out indicated that preferences for different musical dimensions were not only the result of personality, but in some cases were driven significantly by age and gender. In this respect, taking into account the *R^2^* changes, these socio-demographic variables presented a greater weight than the personality in the musical dimensions related to Classical and Ethnic Music, Latin Dance Music and Brazilian Music. However, it is necessary to show that the *R^2^* values obtained in the present work were low, so these results have to be taken with caution.

## Conclusion

This work is one of the few studies carried out in Brazil on the relationship between the preference of music styles and the personality of young and adult listeners. It is especially noteworthy that the large sample counted (>1000 participants) is increasing the representativeness and generalization value of its results.

In the Brazilian field, this work provides important data about how different age groups studied, were using music in personality’s aspects that may be of interest to the analysis and, eventually, the exogenous treatment of a socio-affective behavior (personality) of Brazilian people in need of this kind of psychological counseling. In this sense, an outstanding presence of popular-urban music styles has been found, genuinely “Brazilian” (samba, sertanejo, etc.), which apparently differ the listening habits of Brazilian inhabitants, and their relation to personality types. This was compared to those of other countries, but actually referring to values and intrinsic music construction patterns that go beyond the superficial, and that belong to universal compositional elements common to the above-mentioned popular-urban stylistic macro-category. This allows identifying the need for a more cross-cultural research, framed within the context of social psychology and mass communication studies. This will enable to comparatively analyze the binomial musical preference-type of personality from a global perspective, eliminating the apparent barriers that could exist between different cultures and establishing common patterns between variables defining participants of different nationalities (North, 2010).

In short, the work highlights four general conclusions: the participants listen to little musical variety, reducing their usual sound space (their sonosphere) mainly to musical dimensions related to Pop and more sophisticated Brazilian music styles (popular and religious-protestant); there is a tendency toward a lower preference for music, alien to the Brazilian context; Latin, Brazilian, Classical and Ethnic musical styles correlate positively with most of the analyzed personality types, while Rock music shows a negative correlation; musical preferences are driven not only by personality but in some cases they are also driven by socio-demographic variables (i.e., age and gender).

Finally, one limitation of the study is that it used genre-based self-report measures rather than behavioral excerpt-based measures (e.g., data from affective responses to musical excerpts, digital footprints of Facebook likes, or listening behavior from streaming services). Thus, for example, the recent study by Nave et al. (2018) indicates that an active measure of naturally occurring behavior (i.e., Facebook Likes for musical artists) predicted individual differences in personality.

## Ethics Statement

This study was carried out in accordance with the recommendations of Comitê de Ética em Pesquisa da Universidade Federal do Maranhão, Brazil (CEP/UFMA) [Research Ethics Committee of the Federal University of Maranhão, Brazil (REC/FUM)]. The protocol was approved by the Comitê de Ética em Pesquisa da Universidade Federal do Maranhão (Research Ethics Committee of the Federal University of Maranhão). All subjects gave written informed consent in accordance with the Declaration of Helsinki. More information available at http://portais.ufma.br/PortalProReitoria/pppgi/paginas/pagina_estatica.jsf?id=238.

## Author Contributions

LH, JS-Q, and OL shared the conception, design, and the final version of the work. JS-Q and OL contributed mainly to the theoretical part and in revising it critically. LH and JS-Q contributed mainly to methodological questions and data analysis. LH, JS-Q, and OL are jointly accountable for the content of the work, ensuring that all aspects related to accuracy or integrity of the study are investigated and resolved in an appropriate way. LH, JS-Q, and OL shared the internal consistency of the paper.

## Conflict of Interest Statement

The authors declare that the research was conducted in the absence of any commercial or financial relationships that could be construed as a potential conflict of interest.

## Footnotes

1 https://docs.google.com/forms/d/1zeWZNktoTXpNVdLejTMGqdLOsFdE12QM Xsz9aQHLJFM/viewform
