# Supplementary material for: Music Preferences and Personality in Brazilians
Source: Front Psychol. 2018 Aug 21;9:1488. doi: 10.3389/fpsyg.2018.01488 (PMC6113570; doi:10.3389/fpsyg.2018.01488)
Supplement: Supplementary file 1 [file Table_1.DOC]

Supplementary Material

# Music Preferences and Personality in Brazilians

Lucia Herrera*, João F. Soares, Oswaldo Lorenzo

*** Correspondence:** Lucia Herrera: luciaht@ugr.es

Table S1. Rotated Component Matrix in IPIP Big Five Factor Markers (first analysis).

| Item | Factors | | | | |
| --- | --- | --- | --- | --- | --- |
|  | Emotional Stability | Extraversion | Intellect | Agreeableness | Conscientiousness |
| BF1 | -.004 | **.609** | .076 | .024 | -.010 |
| BF2a | .054 | .025 | .089 | **-.274** | -.091 |
| BF3 | -.101 | .180 | .282 | -.015 | **.314** |
| BF4 | **.701** | .095 | -.047 | -.067 | .095 |
| BF5 | .010 | .141 | **.634** | -.032 | .050 |
| BF6 | -.078 | **-.646** | -.001 | -.088 | .082 |
| BF7 | -.093 | .251 | .178 | **.561** | .002 |
| BF8 | .132 | .066 | .116 | -.015 | **-.708** |
| BF9a | -.263b | .068 | .139 | -.023 | **-.305** |
| BF10 | .147 | .059 | **-.589** | -.007 | .028 |
| BF11 | -.195 | **.513** | .035 | .379 | .069 |
| BF12 | **.380** | .146 | -.031 | -.307b | -.113 |
| BF13a | -.012 | -.081 | **.345** | .224 | .297b |
| BF14a | .175b | -.024 | .205 | **.437** | .380 |
| BF15 | .143 | .179 | **.538** | .143 | -.056 |
| BF16a | .276 | **-.278** | -.144 | .151 | -.149 |
| BF17 | .049 | .095 | .065 | **.727** | .047 |
| BF18a | .038 | .236 | .277 | .216 | **.279** |
| BF19 | **-.598** | .275 | -.007 | -.063 | .168 |
| BF20 | .096 | .151 | **-.434** | -.204 | .156 |
| BF21 | .009 | **.647** | .084 | .194 | .112 |
| BF22 | .086 | -.043 | .013 | **-.697** | .081 |
| BF23 | -.112 | .189 | .242 | .072 | **.503** |
| BF24 | **.739** | .003 | -.036 | -.054 | -.049 |
| BF25 | .005 | .228 | **.622** | -.041 | .173 |
| BF26 | .081 | **-.380** | -.256 | -.160 | .009 |
| BF27 | **-.404** | .157 | -.021 | .315b | .085 |
| BF28 | .160 | .054 | .084 | .007 | **-.628** |
| BF29 | **.732** | -.097 | -.032 | -.034 | -.038 |
| BF30 | .128 | -.019 | **-.358** | -.116 | -.010 |
| BF31 | -.145 | **.629** | .103 | .213 | -.039 |
| BF32 | .157 | -.104 | -.023 | **-.658** | -.014 |
| BF33 | .008 | -.057 | .070 | .100 | **.699** |
| BF34 | **.747** | -.081 | .005 | -.048 | -.115 |
| BF35 | -.083 | .138 | **.584** | .044 | .236 |
| BF36 | -.030 | **-.517** | -.096 | .141 | .159 |
| BF37 | -.071 | .103 | .103 | **.463** | .058 |
| BF38 | .276 | -.078 | -.002 | -.046 | **-.558** |
| BF39 | **.784** | -.073 | -.008 | -.063 | -.136 |
| BF40 | .096 | .072 | **.545** | -.153 | -.028 |
| BF41 | -.068 | **.325** | .072 | -.036 | .095 |
| BF42 | .063 | .100 | .144 | **.635** | .083 |
| BF43 | -.072 | -.009 | .058 | .031 | **.664** |
| BF44 | **.807** | .050 | -.031 | -.129 | .042 |
| BF45a | .211 | -.171 | **.264** | .235 | -.100 |
| BF46 | .146 | **-.643** | -.017 | -.062 | .023 |
| BF47 | -.141 | **.468** | .048 | .352b | .120 |
| BF48 | .054 | .077 | .336 | .111 | **.512** |
| BF49 | **.665** | -.266 | .012 | .016 | -.175 |
| BF50 | .020 | .196 | **.663** | .109 | .100 |

Note. (a) The item saturates below .30 in the factor where theoretically should do so. (b) Factor where it should saturate the item to a greater extent.
