# Supplementary material for: Music Preferences and Personality in Brazilians
Source: Front Psychol. 2018 Aug 21;9:1488. doi: 10.3389/fpsyg.2018.01488 (PMC6113570; doi:10.3389/fpsyg.2018.01488)
Supplement: Supplementary file 2 [file Table_2.DOC]

Supplementary Material

# Music Preferences and Personality in Brazilians

Lucia Herrera*, João F. Soares, Oswaldo Lorenzo

*** Correspondence:** Lucia Herrera: luciaht@ugr.es

Table S2. Rotated Component Matrix in IPIP Big Five Factor Markers (second analysis).

| Item | Factors | | | | |
| --- | --- | --- | --- | --- | --- |
|  | Emotional Stability | Extraversion | Intellect | Conscientiousness | Agreeableness |
| BF44 | **.815** | .043 | -.030 | .022 | -.124 |
| BF39 | **.798** | -.079 | -.007 | -.152 | -.050 |
| BF34 | **.767** | -.078 | .002 | -.128 | -.046 |
| BF29 | **.744** | -.095 | -.036 | -.053 | -.037 |
| BF24 | **.734** | .016 | -.039 | -.075 | -.076 |
| BF4 | **.708** | .080 | -.043 | .069 | -.058 |
| BF49 | **.659** | -.252 | .011 | -.202 | .007 |
| BF19 | **-.582** | .259 | -.005 | .204 | -.081 |
| BF6 | -.051 | **-.663** | -.004 | .099 | -.093 |
| BF21 | .009 | **.652** | .080 | .137 | .198 |
| BF46 | .151 | **-.644** | -.037 | .013 | -.101 |
| BF31 | -.146 | **.637** | .104 | -.007 | .217 |
| BF1 | .000 | **.620** | .087 | .024 | .024 |
| BF36 | -.015 | **-.542** | -.104 | .162 | .117 |
| BF11 | -.186 | **.521** | .022 | .100 | .379 |
| BF26 | .091 | **-.381** | -.254 | .027 | -.204 |
| BF41 | -.066 | **.323** | .083 | .117 | -.036 |
| BF50 | .021 | .189 | **.661** | .109 | .104 |
| BF5 | .028 | .123 | **.649** | .075 | .006 |
| BF25 | .020 | .210 | **.636** | .184 | -.044 |
| BF10 | .155 | .080 | **-.611** | .044 | -.056 |
| BF35 | -.064 | .112 | **.581** | .255 | .086 |
| BF40 | .098 | .067 | **.558** | -.017 | -.124 |
| BF15 | .144 | .180 | **.530** | -.047 | .128 |
| BF20 | .111 | .168 | **-.440** | .180 | -.247 |
| BF30 | .117 | .000 | **-.367** | -.013 | -.146 |
| BF33 | .034 | -.064 | .053 | **.722** | .083 |
| BF8 | .123 | .077 | .107 | **-.698** | -.016 |
| BF43 | -.048 | -.030 | .045 | **.693** | .036 |
| BF28 | .149 | .060 | .083 | **-.632** | -.008 |
| BF38 | .262 | -.058 | -.007 | **-.549** | -.042 |
| BF23 | -.086 | .161 | .257 | **.519** | .080 |
| BF48 | .073 | .053 | .328 | **.519** | .119 |
| BF3 | -.076 | .159 | .288 | **.345** | .003 |
| BF22 | .081 | -.012 | .022 | .070 | **-.746** |
| BF17 | .072 | .076 | .038 | .094 | **.734** |
| BF32 | .155 | -.075 | -.015 | -.025 | **-.708** |
| BF42 | .070 | .071 | .122 | .109 | **.637** |
| BF7 | -.092 | .233 | .166 | .028 | **.607** |
| BF37 | -.052 | .093 | .089 | .103 | **.472** |
