# Supplementary material for: Music Preferences and Personality in Brazilians
Source: Front Psychol. 2018 Aug 21;9:1488. doi: 10.3389/fpsyg.2018.01488 (PMC6113570; doi:10.3389/fpsyg.2018.01488)
Supplement: Supplementary file 3 [file Table_3.DOC]

Supplementary Material

# Music Preferences and Personality in Brazilians

Lucia Herrera*, João F. Soares, Oswaldo Lorenzo

*** Correspondence:** Lucia Herrera: luciaht@ugr.es

Table S3. List of musical styles.

| 1. Alternative | 38. Gregorian |
| --- | --- |
| 1. Arrocha | 39. Medieval |
| 1. Axé-Music | 40. Renaissance |
| 1. Blues | 41. Baroque |
| 1. Bolero | 42. Classicism |
| 1. Bossa-Nova | 43. Romanticism |
| 1. Brega | 44. Impressionist |
| 1. Chorinho | 45. Nationalist |
| 1. Country | 46. Vanguard |
| 1. Dance | 47. Contemporary |
| 1. Disco | 48. New Age |
| 1. Electronic | 49. Pagode |
| 1. Emocore | 50. Pop |
| 1. Fado | 51. Pop Rock |
| 1. Flamenco | 52. Punk |
| 1. Folkloric | 53. Rap |
| 1. Folk | 54. Reggae |
| 1. Forró | 55. Reggaeton |
| 1. Frevo | 56. Rhythm and Blues |
| 1. Funk | 57. Rock |
| 1. Funk Carioca | 58. Progressive Rock |
| 1. Gospel | 59. Romantic |
| 1. Gothic | 60. Rumba |
| 1. Grunge | 61. Salsa |
| 1. Hard Rock | 62. Samba |
| 1. Hardcore | 63. Sertanejo |
| 1. Heavy Metal | 64. Sertanejo Universitário |
| 1. Hip-Hop | 65. Ska |
| 1. House | 66. Soul |
| 1. Indie | 67. Surf Music |
| 1. Instrumental and/or Soundtracks | 68. Swingueira |
| 1. Jazz | 69. Tango |
| 1. Lambada | 70. Tecno |
| 1. Mambo | 71. Tecno Brega |
| 1. Merengue | 72. Trance |
| 1. Brazilian Popular Music | 73. Waltz |
| 1. Ethnic | 74. Others |
