# Supplementary material for: Music Preferences and Personality in Brazilians
Source: Front Psychol. 2018 Aug 21;9:1488. doi: 10.3389/fpsyg.2018.01488 (PMC6113570; doi:10.3389/fpsyg.2018.01488)
Supplement: Supplementary file 4 [file Table_4.DOC]

Supplementary Material

# Music Preferences and Personality in Brazilians

Lucia Herrera*, João F. Soares, Oswaldo Lorenzo

*** Correspondence:** Lucia Herrera: luciaht@ugr.es

Table S4. Rotated Component Matrix in the Questionnaire on Musical Style Preferences.

| Musical Styles | Factors | | | | | | | | | | |
| --- | --- | --- | --- | --- | --- | --- | --- | --- | --- | --- | --- |
|  | CEM | RM | BMM | LDM | BM | EM | AM | ALM | AAM | PM | UM |
| Baroque | **.881** | -.035 | -.145 | .063 | .060 | .011 | .061 | -.084 | -.121 | -.096 | -.031 |
| Romanticism | **.880** | -.043 | -.128 | .055 | .012 | -.039 | .036 | -.125 | -.065 | -.055 | .017 |
| Classicism | **.867** | -.047 | -.150 | .073 | .037 | -.023 | .075 | -.125 | -.092 | -.058 | .002 |
| Impressionist | **.859** | -.027 | -.094 | .126 | .019 | -.023 | .052 | -.025 | -.032 | -.053 | -.076 |
| Renaissance | **.853** | .030 | -.103 | .085 | .071 | .014 | .051 | -.014 | -.112 | -.066 | -.005 |
| Nationalist | **.782** | -.042 | .022 | .094 | .036 | -.105 | -.011 | .005 | .070 | .051 | .051 |
| Medieval | **.772** | .169 | -.106 | .085 | .112 | .075 | -.023 | .042 | -.063 | -.092 | .071 |
| Vanguard | **.724** | .013 | .046 | .078 | .088 | -.089 | .099 | .221 | .118 | .087 | -.018 |
| Gregorian | **.723** | .061 | -.051 | .196 | .179 | .060 | -.007 | .054 | -.112 | -.067 | .038 |
| Contemporary | **.641** | .019 | .044 | -.007 | .028 | -.015 | .141 | .185 | .092 | .143 | -.009 |
| Ethnic | **.488** | .055 | -.024 | .218 | .287 | .024 | .047 | .369 | .080 | -.078 | -.151 |
| Waltz | **.479** | -.037 | .027 | .261 | .306 | .074 | -.086 | -.138 | -.003 | -.047 | .205 |
| Tango | **.417** | .004 | -.027 | .389 | .391 | .070 | -.031 | -.050 | .057 | -.185 | .067 |
| Flamenco | **.409** | .030 | -.052 | .400 | .315 | .057 | .062 | -.004 | -.027 | -.207 | -.032 |
| Fado | **.361** | .004 | -.068 | .357 | .345 | .094 | .001 | .178 | -.151 | -.124 | -.175 |
| Hard Rock | -.018 | **.864** | -.002 | -.018 | -.066 | .069 | .124 | .044 | .036 | .044 | .012 |
| Heavy Metal | .040 | **.853** | -.031 | -.052 | -.058 | .090 | .032 | -.093 | .033 | -.033 | .019 |
| Hardcore | -.044 | **.810** | -.012 | -.018 | -.061 | .089 | .105 | .176 | .130 | -.025 | .003 |
| Grunge | -.025 | **.709** | -.087 | -.026 | -.031 | .072 | .127 | .353 | .045 | -.017 | -.163 |
| Progressive Rock | .128 | **.706** | -.139 | .030 | .082 | .058 | .252 | .079 | .050 | .118 | -.086 |
| Rock | -.073 | **.694** | -.042 | -.065 | .115 | .076 | .164 | .057 | .082 | .395 | -.027 |
| Punk | -.012 | **.687** | .000 | .003 | -.060 | .124 | .083 | .252 | .227 | .024 | -.031 |
| Gothic | .075 | **.550** | .017 | .010 | -.104 | .231 | -.041 | .109 | -.023 | -.070 | .242 |
| Emocore | -.034 | **.461** | -.004 | -.062 | -.194 | .082 | -.041 | .435 | .091 | -.058 | .082 |
| Ska | -.018 | **.367** | -.002 | -.018 | -.066 | .069 | .124 | .044 | .036 | .044 | .012 |
| Arrocha | -.164 | -.027 | **.765** | -.032 | -.073 | .091 | .007 | -.032 | .079 | .036 | .099 |
| Pagode | -.091 | -.149 | **.721** | .066 | .107 | .018 | -.044 | -.082 | .175 | .165 | -.014 |
| University Sertanejo | -.173 | -.131 | **.717** | -.136 | -.064 | .087 | -.151 | -.073 | .052 | .150 | .246 |
| Axé-Music | -.094 | -.085 | **.716** | .116 | .090 | .103 | -.050 | -.096 | .030 | .133 | .035 |
| Sertanejo | -.075 | -.058 | **.703** | -.060 | .132 | .040 | -.116 | -.046 | .046 | .112 | .275 |
| Brega | -.057 | .087 | **.644** | .157 | .229 | .146 | -.054 | .019 | -.061 | -.107 | .040 |
| Forró | .008 | -.159 | **.623** | .185 | .344 | -.004 | -.012 | -.094 | .039 | .132 | .024 |
| Tecno Brega | -.046 | .066 | **.606** | .142 | .003 | .267 | .024 | .140 | -.029 | -.040 | -.073 |
| Swingueira | .024 | .056 | **.580** | .176 | -.148 | -.031 | .156 | .050 | .030 | .039 | .055 |
| Funk Carioca | -.030 | -.054 | **.555** | -.011 | -.041 | .201 | .010 | .026 | .404 | -.029 | -.164 |
| Funk | .039 | .127 | **.439** | .076 | .113 | .076 | .367 | .002 | .351 | -.033 | -.093 |
| Merengue | .149 | -.008 | .200 | **.813** | .043 | .051 | .103 | -.004 | .073 | .124 | .076 |
| Mambo | .167 | -.020 | .121 | **.776** | .093 | .096 | .153 | .016 | .079 | .071 | .043 |
| Rumba | .245 | -.043 | .030 | **.771** | .107 | .056 | .156 | .072 | .046 | .011 | -.091 |
| Salsa | .231 | -.056 | .101 | **.731** | .157 | .016 | .160 | -.026 | .065 | .021 | .073 |
| Lambada | .013 | -.015 | .464 | **.523** | .086 | .093 | -.027 | -.038 | .012 | -.003 | .216 |
| Frevo | .297 | -.058 | .231 | **.436** | .424 | -.042 | .112 | -.030 | .044 | .018 | -.066 |
| Brazilian Popular Music | .037 | -.102 | .136 | -.035 | **.709** | -.088 | .155 | .100 | .064 | .332 | .015 |
| Bossa-Nova | .220 | -.098 | -.015 | .082 | **.663** | -.034 | .326 | .000 | .005 | .083 | .086 |
| Samba | .123 | -.096 | .317 | .157 | **.661** | -.105 | .160 | .068 | .196 | .097 | -.156 |
| Chorinho | .364 | -.053 | .000 | .196 | **.644** | -.042 | .206 | -.107 | -.020 | -.070 | .042 |
| Bolero | .175 | .017 | .187 | .362 | **.483** | .081 | .048 | -.100 | -.034 | -.145 | .252 |
| Folkloric | .386 | -.031 | .133 | .320 | **.444** | -.048 | -.090 | .168 | .043 | -.081 | .061 |
| Dance | -.121 | .040 | .250 | .034 | .001 | **.709** | -.058 | -.006 | .083 | .268 | .243 |
| Electronic | -.047 | .116 | .183 | -.072 | -.106 | **.692** | -.045 | .065 | .277 | .088 | .166 |
| Techno | .009 | .173 | .246 | .143 | .030 | **.692** | .013 | -.019 | .064 | .053 | -.067 |
| Disco | .022 | .127 | .042 | .173 | .089 | **.624** | .132 | .056 | .031 | .206 | .102 |
| House | -.027 | .167 | .085 | .062 | -.143 | **.592** | .202 | .230 | .155 | -.012 | -.067 |
| Trance | .085 | .241 | .099 | -.060 | -.101 | **.519** | .173 | .132 | .068 | -.041 | -.261 |
| New Age | .278 | .239 | -.146 | .197 | .138 | **.323** | .039 | .256 | -.128 | .135 | -.075 |
| Soul | .045 | .212 | .004 | .202 | .185 | .095 | **.655** | .099 | .072 | .080 | .083 |
| Jazz | .334 | .095 | -.095 | .198 | .363 | .011 | **.625** | .021 | .033 | -.079 | .161 |
| Blues | .120 | .291 | -.077 | .067 | .331 | .056 | **.619** | .021 | -.003 | -.067 | .221 |
| Rhythm and Blues | .085 | .254 | -.111 | .208 | .080 | .168 | **.613** | .130 | .050 | .107 | .006 |
| Surf Music | -.024 | .336 | .127 | .069 | .024 | .116 | **.456** | .351 | .050 | .101 | -.174 |
| Alternative | -.029 | .304 | .031 | -.023 | .066 | .117 | .072 | **.637** | .155 | .081 | .038 |
| Indie | -.032 | .430 | -.140 | -.099 | -.099 | .167 | .191 | **.627** | .039 | .008 | -.043 |
| Folk | .202 | .333 | -.182 | .107 | .151 | .116 | .150 | **.571** | -.013 | -.077 | .112 |
| Rap | -.062 | .201 | .112 | .066 | .092 | .160 | .016 | .126 | **.803** | .013 | .001 |
| Hip-Hop | -.135 | .197 | .104 | .064 | .051 | .250 | .039 | .041 | **.761** | .058 | .101 |
| Reggae | -.105 | .195 | .320 | .140 | .286 | .013 | .211 | .064 | **.441** | .180 | .008 |
| Reggaeton | -.006 | .195 | .295 | .272 | -.159 | .125 | .156 | .117 | **.387** | -.025 | .018 |
| Pop Rock | -.120 | .206 | .198 | .011 | .048 | .251 | .069 | -.030 | .036 | **.782** | .123 |
| Pop | -.096 | .033 | .229 | .067 | .024 | .333 | .051 | .002 | .062 | **.733** | .122 |
| Gospel | -.036 | -.195 | .203 | .002 | -.093 | -.063 | .037 | -.062 | .060 | .079 | **.642** |
| Country | .031 | .175 | .230 | .084 | .085 | .157 | .113 | .088 | -.018 | .112 | **.510** |
| Romantic | -.127 | -.052 | .304 | .122 | .156 | .037 | -.113 | .038 | -.013 | .451 | **.454** |
| Instrumental and/or Soundtracks | .313 | .092 | -.052 | .093 | .116 | .064 | .231 | .025 | .029 | .043 | **.435** |

Note. CEM = Classical and Ethnic Music, RM = Rock Music, BMM = Brazilian Mainstream Music, LDM = Latin Dance Music, BM = Brazilian Music, EM = Electronic Music, AM = American Music, ALM = Alternative Music, AAM = Afro-American Music, PM = Pop Music, UM = Upbeat Music
