# Supplementary material for: Music Preferences and Personality in Brazilians
Source: Front Psychol. 2018 Aug 21;9:1488. doi: 10.3389/fpsyg.2018.01488 (PMC6113570; doi:10.3389/fpsyg.2018.01488)
Supplement: Supplementary file 5 [file Table_5.DOC]

Supplementary Material

# Music Preferences and Personality in Brazilians

Lucia Herrera*, João F. Soares, Oswaldo Lorenzo

*** Correspondence:** Lucia Herrera: luciaht@ugr.es

Table S5. Raw Data Eigenvalues, Mean and Percentile Random Data Eigenvalues (Parallel Analysis).

| Root | Raw Data | Mean | Percentile |
| --- | --- | --- | --- |
| 1.00 | 11.986 | 1.559 | 1.599 |
| 2..0 | 9.529 | 1.518 | 1.550 |
| 3.00 | 8.002 | 1.487 | 1.515 |
| 4.00 | 3.195 | 1.460 | 1.485 |
| 5.00 | 2.476 | 1.435 | 1.458 |
| 6.00 | 2.234 | 1.414 | 1.435 |
| 7.00 | 1.828 | 1.393 | 1.413 |
| 8.00 | 1.487 | 1.373 | 1.393 |
| 9.00 | 1.356 | 1.355 | 1.373 |
| 10.00 | 1.325 | 1.337 | 1.356 |
| 11.00 | 1.276 | 1.320 | 1.338 |
| 12.00 | 1.173 | 1.304 | 1.320 |
| 13.00 | 1.017 | 1.288 | 1.304 |
| 14.00 | .998 | 1.271 | 1.288 |
| 15.00 | .941 | 1.256 | 1.271 |
| 16.00 | .912 | 1.241 | 1.257 |
| 17.00 | .872 | 1.227 | 1.241 |
| 18.00 | .840 | 1.212 | 1.226 |
| 19.00 | .790 | 1.198 | 1.212 |
| 20.00 | .759 | 1.185 | 1.199 |
| 21.00 | .748 | 1.171 | 1.185 |
| 22.00 | .691 | 1.158 | 1.171 |
| 23.00 | .658 | 1.144 | 1.158 |
| 24.00 | .638 | 1.131 | 1.144 |
| 25.00 | .617 | 1.119 | 1.132 |
| 26.00 | .612 | 1.106 | 1.119 |
| 27.00 | .580 | 1.094 | 1.107 |
| 28.00 | .568 | 1.082 | 1.095 |
| 29.00 | .557 | 1.070 | 1.082 |
| 30.00 | .537 | 1.058 | 1.070 |
| 31.00 | .535 | 1.046 | 1.058 |
| 32.00 | .518 | 1.034 | 1.046 |
| 33.00 | .493 | 1.023 | 1.035 |
| 34.00 | .485 | 1.011 | 1.023 |
| 35.00 | .479 | 1.000 | 1.012 |
| 36.00 | .465 | .988 | 1.000 |
| 37.00 | .457 | .977 | .990 |
| 38.00 | .450 | .966 | .978 |
| 39.00 | .443 | .955 | .967 |
| 40.00 | .434 | .944 | .955 |
| 41.00 | .424 | .933 | .945 |
| 42.00 | .411 | .922 | .934 |
| 43.00 | .405 | .912 | .923 |
| 44.00 | .399 | .901 | .912 |
| 45.00 | .385 | .890 | .901 |
| 46.00 | .372 | .880 | .891 |
| 47.00 | .360 | .869 | .880 |
| 48.00 | .355 | .858 | .869 |
| 49.00 | .346 | .847 | .858 |
| 50.00 | .341 | .837 | .848 |
| 51.00 | .323 | .826 | .838 |
| 52.00 | .317 | .816 | .827 |
| 53.00 | .308 | .806 | .817 |
| 54.00 | .301 | .795 | .806 |
| 55.00 | .298 | .784 | .796 |
| 56.00 | .282 | .774 | .786 |
| 57.00 | .272 | .764 | .775 |
| 58.00 | .269 | .753 | .765 |
| 59.00 | .261 | .742 | .753 |
| 60.00 | .257 | .731 | .742 |
| 61.00 | .244 | .720 | .732 |
| 62.00 | .230 | .709 | .720 |
| 63.00 | .225 | .698 | .710 |
| 64.00 | .223 | .687 | .699 |
| 65.00 | .203 | .675 | .688 |
| 66.00 | .196 | .663 | .676 |
| 67.00 | .186 | .651 | .665 |
| 68.00 | .176 | .639 | .652 |
| 69.00 | .169 | .626 | .638 |
| 70.00 | .156 | .612 | .626 |
| 71.00 | .129 | .597 | .612 |
| 72.00 | .091 | .580 | .596 |
| 73.00 | .086 | .558 | .578 |

Note. Specifications for this Run: Ncases = 1050, Nvars = 73, Ndatsets = 2100, Percent = 95
